# Thyroid Teratoma in a Pediatric Patient

**DOI:** 10.7759/cureus.29395

**Published:** 2022-09-21

**Authors:** Meera R Laxman, Laura L Hayes, Santino S Cervantes, Tamarah Westmoreland

**Affiliations:** 1 Pediatric Surgery, Nemours Children's Hospital, Orlando, USA; 2 Pediatric Radiology, Nemours Children's Hospital, Pensacola, USA; 3 Pediatric Otolaryngology, Nemours Children's Hospital, Orlando, USA; 4 Pediatric Surgery, University of Central Florida College of Medicine, Orlando, USA

**Keywords:** pediatric teratoma, cervical teratoma, thyroid pathologies, head and neck tumor, pediatric surgery, thyroid teratoma

## Abstract

Congenital thyroid teratomas are rare in the pediatric population as well as in the adult population. While they are typically found in the gonadal regions, extragonadally, they are commonly found in the sacrococcygeal region, with teratomas of the head and neck rarely found, comprising only about 1%-6% of all pediatric teratomas. Due to a concern for potential airway compromise and increased risk of malignancy with age, early surgical excision is recommended. In this case report, we present a two-year-old female who underwent laryngoscopy with subsequent right thyroid lobectomy for a large thyroid mass, which was found to be a congenital thyroid teratoma.

## Introduction

Thyroid teratomas are rare in both pediatric and adult populations [1]. Teratomas are commonly found in gonadal regions, involving ovaries or testicles, or extragonadal regions, involving the sacrococcygeal region, most commonly mediastinal, gastric, retroperitoneal, intracranial, cervical, and craniofacial regions [2]. They are seldom found in the neck region, composing only about 1%-6% of all types of pediatric teratomas, and very few are located entirely within the thyroid gland [2-4]. Within the head and neck region, cervical teratomas are most common, primarily identified on antenatal ultrasound [2]. In almost all cases, these cervical teratomas are found to be benign [4]. However, due to the possibility of extension into the mediastinum or displacement of the trachea, there can be a concern for morbidity and mortality related to respiratory compromise from airway obstruction [2]. As such, surgical intervention is often required. While malignancy is estimated to be around 5% for cervical teratomas, earlier surgical intervention is also favored due to the increased risk of malignancy with advancing age as this tumor can grow fairly quickly in the younger population [1,2]. Therefore, surgical excision of the involved thyroid lobe is highly recommended.

## Case presentation

An otherwise healthy two-year-old female presented to the general surgery clinic for evaluation of a right neck cyst, initially seen on fetal ultrasound (US) in Mexico. After she was born, the mother's gynecologist reported the mass was normal and did not recommend further follow-up for the mother. While the patient remained asymptomatic, the mother noticed the mass continued to grow. Per her mother, the cyst involved her right shoulder; however, she had normal use of her right hand without any apparent weakness or difficulty using her right arm. Upon initial physical exam, the thyroid had palpable fullness, but there were no other concerning physical exam findings.

**Figure 1:**
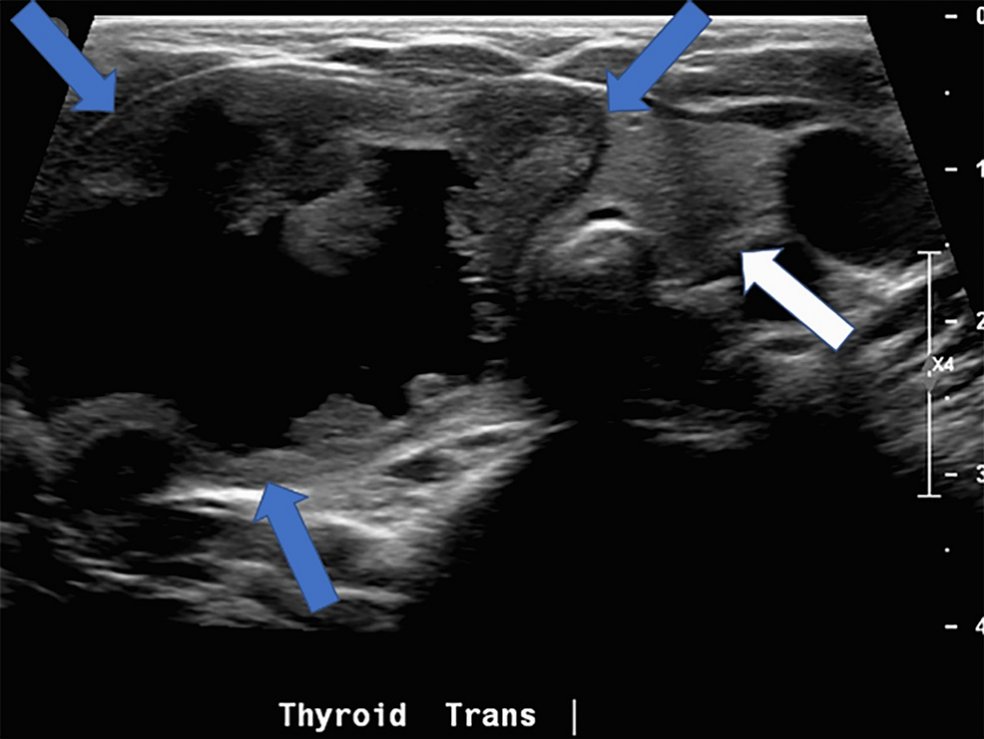
Transverse ultrasound image of the thyroid gland demonstrates a circumscribed, solid mass containing a hypoechoic, cystic component with irregular walls confined to the right lobe (blue arrows). The left lobe of the thyroid gland appears normal (white arrow). Image courtesy: This image was provided by Dr. Laura L. Hayes from the radiology department at Nemours Children’s Health System.

**Figure 2:**
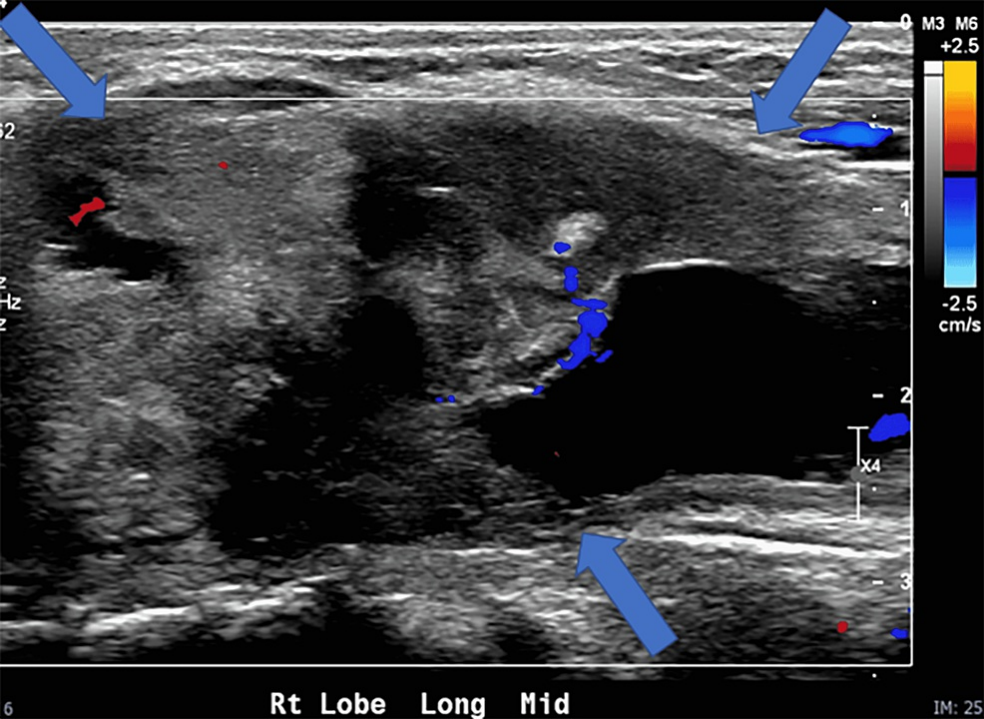
Longitudinal ultrasound image with color Doppler demonstrates the heterogeneous echotexture of the solid and cystic mass in the right lobe of the thyroid gland (blue arrows). Image courtesy: This image was provided by Dr. Laura L. Hayes from the radiology department at Nemours Children’s Health System.

Repeat US of the neck was performed, which showed a complex, cystic, and solid mass (5.7 cm x 2 cm x 4 cm) confined to the right lobe of the thyroid gland (Figures 1, 2). The appearance was concerning for possible congenital teratoma or primary thyroid tumor as well as a fourth branchial cleft cyst.

**Figure 3:**
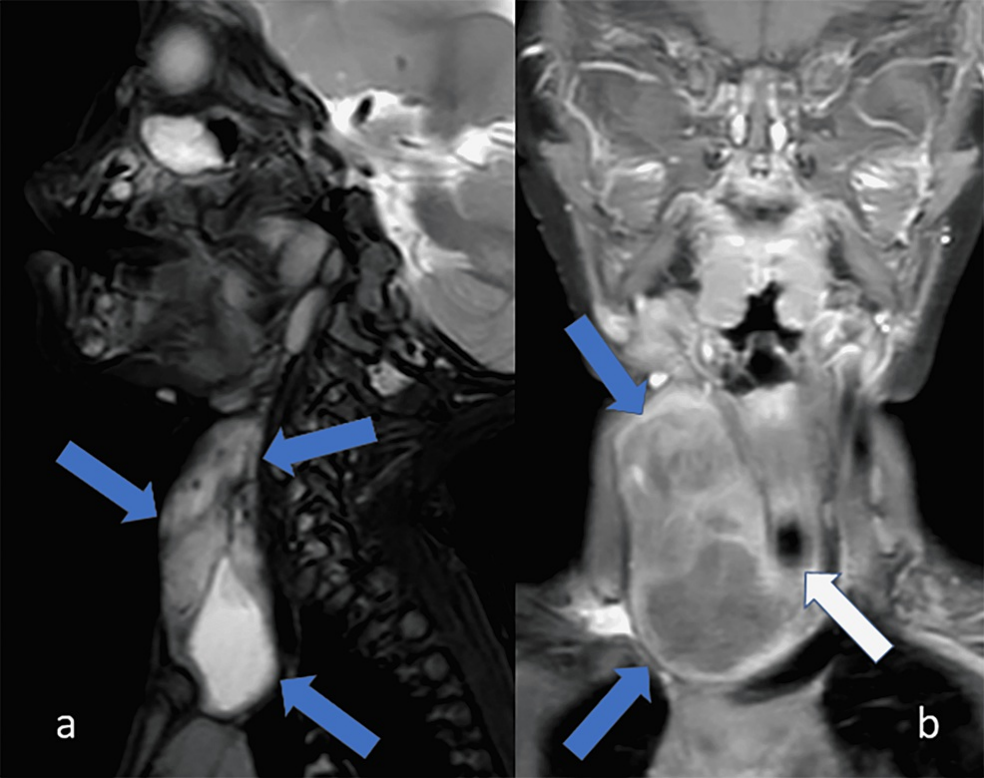
(a) Sagittal T2-weighted image with fat saturation demonstrates the large, heterogeneous lesion extending from the mid cervical region to the thoracic inlet (blue arrows). (b) Coronal post-contrast, T1-weighted image with fat saturation demonstrates the partially enhancing, cystic-appearing lesion in the thyroid gland (blue arrows) displacing the trachea to the left (white arrow). Image courtesy: This image was provided by Dr. Laura L. Hayes from the radiology department at Nemours Children’s Health System.

US was followed by magnetic resonance imaging (MRI) of the neck with and without contrast (Figure 3). MRI demonstrated a large cystic and solid-appearing mass-like lesion in the right neck involving the right lobe of the thyroid gland and producing a local mass effect including some bowing of the trachea to the left without significant narrowing. There were also some mildly prominent lymph nodes present in the right neck and submandibular region. The left lobe of the thyroid gland appeared normal. At this point, a fourth branchial cleft cyst with possible fistulous tract communicating with the right piriform sinus was considered a leading differential, with thyroid teratoma considered less likely.

**Figure 4:**
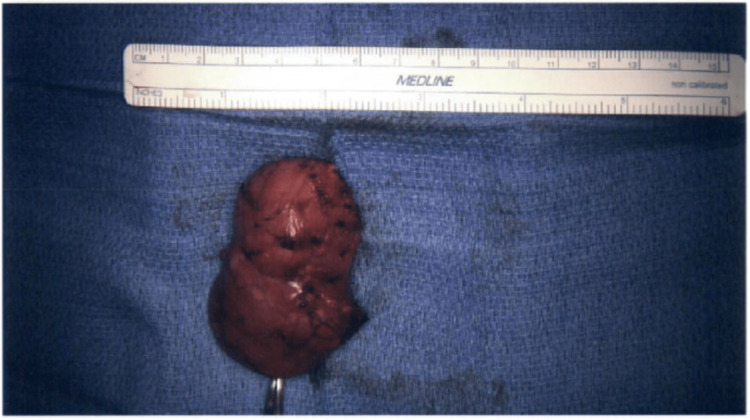
Surgical specimen

On the day of surgery, the right thyroid mass was still palpable, but there were no other significant physical exam findings. The patient was taken to the operating room (OR) for a right thyroid lobectomy with a laryngoscopy. In the OR, the patient was initially intubated with a 4.5-cuffed endotracheal tube, and suspension laryngoscopy, with the Parsons #3 laryngoscope, was performed to look for a defect in the right piriform sinus. No defect was seen. The endotracheal tube was replaced with a 5-0 Medtronic Nerve Integrity Monitoring (NIM) endotracheal tube, and electrodes were placed at the level of the cricopharyngeus muscle. The patient was then prepped for neck exploration. At approximately two fingerbreadths above the sternal notch, an 8-cm incision was made down through the subcutaneous tissue. Platysmal flaps were raised superiorly to the thyroid cartilage and inferiorly to the sternal notch. Strap muscles were then divided in the midline vertically, kept intact, and dissected off of the thyroid gland. The right neck mass was found to be contained completely within the right thyroid lobe. The right-sided middle thyroid vein was identified and ligated with a #3-0 Vicryl suture and sharply divided. The right recurrent laryngeal nerve was identified visually and protected. Next, the vessels to the right superior pole of the thyroid gland were divided utilizing a bipolar. Then, the isthmus was dissected off the trachea with the bipolar and divided off of the left thyroid lobe using electrocautery. No masses were visualized or palpated on the left thyroid lobe. The right thyroid lobe was removed and passed off as a specimen for pathology (Figure 4). The right upper parathyroid glands were viable, and no right lower parathyroid gland was visualized.

Postoperatively, the patient did well. Postoperative management required a multidisciplinary approach involving endocrinology and otolaryngology. Otolaryngology was primarily involved intraoperatively to assist with the removal of the teratoma as it was close to the airway, and endocrinology monitored the patient's labs postoperatively. Initial postoperative labs, five hours after surgery, showed mild hypocalcemia, likely due to transient hypoparathyroidism. On postoperative day (POD) #1, calcium normalized after implementing endocrine recommendations of 400 mg calcium carbonate four times a day with meals. On POD #2, calcium supplementation was decreased to 400 mg three times a day with meals. All labs remained within normal limits on POD #2, 4, and 17. The patient was discharged home on POD #2 in stable condition, has been followed by endocrinology, and has been doing well overall. Over the past two years, her thyroid stimulating hormone (TSH) and free T4 have been within normal limits. Her parathyroid hormone levels have been slightly decreased, which is expected with a partial thyroidectomy, but she has been doing well at her follow-up visits.

## Discussion

The thyroid is located in the neck and is an endocrine gland that primarily functions to both formulate and secrete thyroid hormones and maintain iodine levels within the body. These hormones are thyroxine (T4) and triiodothyronine (T3) [5].

Pathology showed, via microscopic examination, that the right lobe of the thyroid gland was almost entirely replaced by a teratoma. All three mature germ cell elements were present. The predominant element was identified to be glial. No immature or malignant germ cell elements were seen. The tumor did not extend beyond the surgical margin. Three small benign lymph nodes were also present and negative for involvement by tumor. The inferior aspect of the right thyroid lobe showed a large cystic cavity with aggregates of histiocytic-appearing cells and some containing brown-tan pigment consistent with hemosiderin as well as squamoid-appearing cells and small granulomas that suggest rupture of an epidermal inclusion cyst.

Although thyroid teratomas are rare in the pediatric population [1], a very similar case was presented in the way our patient presented. An 11-month-old infant male was also found to have a thyroid mass on the right lobe present since birth, histologically similar to our patient, where all three primordial germ layers were involved, which was found to be a benign thyroid teratoma [6]. Another similar case of thyroid teratoma in a newborn was identified antenatally, which also involved all three germ cell layers and was found to be benign, just as in our case [4]. More recently, there is a case report of a newborn girl who presented with respiratory distress from a lesion on the right lobe of her thyroid requiring surgery on the 13th day of life. She recovered well after surgery and was found to be free of recurrence at one-year post-excision [7]. Similarly, a mature thyroid teratoma was excised from a neonate at just five-days old after detection on prenatal imaging. The teratoma had histopathologic similarities to those found in our patient including three germ cell layers without immature components to suggest malignancy [8]. Additionally, mature teratomas appear to be the most common type and histologically were found in children as found in our patient [2]. They tend to be benign in almost all cases involving children but locally aggressive [4]. Additionally, based on the review of literature, most reported cases of thyroid teratomas in children appear to present within just one lobe versus involving the entire thyroid gland [4,7,9]. Although they can present with varying degrees of respiratory distress, they can also present asymptomatically as in our patient. These tumors also appear to present without pain in infants and children but appear to have painful cystic components in later adolescence and young adulthood as seen in three different cases of patients presented at ages 15, 13, and 23 years [10]. After surgery, our patient’s remaining thyroid was functioning normally.

## Conclusions

While uncommon in adolescence as well as adulthood, it seems apparent that thyroid teratomas tend to present similarly in pediatric patients. They are typically benign, involve mixed cystic as well as solid components, and require surgical excision prior to the potential for airway compromise and the increased risk of malignancy with age. As such, diagnosis prenatally can be crucial in a majority of cases. Most cases of congenital thyroid teratoma are identified via fetal ultrasound. Surgical excision is highly favored to prevent respiratory issues or malignancy. Therefore, partial thyroid lobectomy of the involved lobe is recommended.
